# Prebiotic Driven Increases in IL-17A Do Not Prevent *Campylobacter jejuni* Colonization of Chickens

**DOI:** 10.3389/fmicb.2019.03030

**Published:** 2020-01-14

**Authors:** Geraldine M. Flaujac Lafontaine, Philip J. Richards, Phillippa L. Connerton, Peter M. O’Kane, Nacheervan M. Ghaffar, Nicola J. Cummings, Neville M. Fish, Ian F. Connerton

**Affiliations:** ^1^Division of Microbiology, Brewing and Biotechnology, School of Biosciences, University of Nottingham, Loughborough, United Kingdom; ^2^Saputo Dairy UK, Dairy Crest Innovation Centre, Harper Adams University, Newport, United Kingdom

**Keywords:** *Campylobacter*, galacto-oligosaccharide, prebiotic, broiler chicken, innate immunity, microbiota, Th17, pro-inflammatory response

## Abstract

Worldwide *Campylobacter jejuni* is a leading cause of foodborne disease. Contamination of chicken meat with digesta from *C. jejuni*-positive birds during slaughter and processing is a key route of transmission to humans through the food chain. Colonization of chickens with *C. jejuni* elicits host innate immune responses that may be modulated by dietary additives to provide a reduction in the number of campylobacters colonizing the gastrointestinal tract and thereby reduce the likelihood of human exposure to an infectious dose. Here we report the effects of prebiotic galacto-oligosaccharide (GOS) on broiler chickens colonized with *C. jejuni* when challenged at either an early stage in development at 6 days of age or 20 days old when campylobacters are frequently detected in commercial flocks. GOS-fed birds had increased growth performance, but the levels of *C. jejuni* colonizing the cecal pouches were unchanged irrespective of the age of challenge. Dietary GOS modulated the immune response to *C. jejuni* by increasing cytokine IL-17A expression at colonization. Correspondingly, reduced diversity of the cecal microbiota was associated with *Campylobacter* colonization in GOS-fed birds. In birds challenged at 6 days-old the reduction in microbial diversity was accompanied by an increase in the relative abundance of *Escherichia* spp. Whilst immuno-modulation of the Th17 pro-inflammatory response did not prevent *C. jejuni* colonization of the intestinal tract of broiler chickens, the study highlights the potential for combinations of prebiotics, and specific competitors (synbiotics) to engage with the host innate immunity to reduce pathogen burdens.

## Introduction

*Campylobacter* spp. are recognized as the major contributor to bacterial foodborne illness worldwide (Kaakoush et al., 2015). Campylobacterosis was the most frequently reported human zoonotic disease in the European Union in 2017 with 246,158 confirmed cases of gastrointestinal illness (EFSA, 2018). The most common species associated with human disease is *C. jejuni* (84.4%), but *C. coli* also represent a significant disease burden (9.2%; EFSA, 2018). *C. jejuni* and *C. coli* are referred to as thermophilic species as they can grow at 42°C, making them suited to colonize the intestinal tracts of poultry species (reviewed by Sahin et al., 2015). Poultry are a major source of campylobacters with an estimated 80% of human illness arising from poultry sources (Andreoletti et al., 2010). Source attribution estimates referenced at the point of exposure indicate 65–69% of human cases are from exposure to chicken meat (Ravel et al., 2017). Poultry meat is frequently contaminated with intestinal content harboring high levels of *Campylobacter* cells during slaughter and carcass processing, which constitutes the main risk to public health (Osimani et al., 2017). This has prompted the EU to adopt a microbiological sampling plan for broiler chicken carcasses with a limit of 1,000 CFU/g (Commission Regulation (EU) 2017/1495). Strict on-farm biosecurity measures to prevent *Campylobacter* exposure and flock colonization of broiler chickens have been implemented in many countries, but these alone do not maintain *Campylobacter*-free flocks (Newell et al., 2011). Intervention strategies have been developed aimed at reducing levels of *Campylobacter* colonization, and thereby human exposure, if the reductions can be translated on to chicken meat (Rosenquist et al., 2003; Newell et al., 2011; Sahin et al., 2015). *Campylobacter* colonization has been associated with poor flock health and performance in commercial broiler chicken production (Bull et al., 2008), although performance issues are not manifest in all circumstances (Gormley et al., 2014). The impact of *Campylobacter* colonization on bird health has been reported to vary with the broiler breed/rate of growth, stocking density, intercurrent infectious or immunosuppressive challenges and the colonizing organism (Humphrey et al., 2015; Li L. et al., 2018). It is, however, clear that *Campylobacter* colonization elicits a Th17 pro-inflammatory response in broiler chickens (Reid et al., 2016; Connerton et al., 2018). Intestinal intraepithelial lymphocytes characterized as either CD3^+^CD25^+^ cells or γδ T cells have been reported to express intracellular IL-17A in the lower intestine of chickens (Walliser and Göbel, 2018). Not all inflammatory responses lead to negative outcomes for the host. It has been questioned whether dietary anti-inflammatory additives aimed at the detrimental consequences of intestinal inflammation may actually impair necessary responses of young animals that are required to overcome the challenges present in commercial production to achieve favorable performance outcomes (Broom and Kogut, 2018). Zootechnical performance remains a key driver in the poultry industry, at the same time public and regulatory concerns are mounting regarding welfare and antibiotic use in poultry production. Although progress has been made toward reducing antibiotic use in poultry production in several countries has been reported more remains to be achieved (Speksnijder et al., 2015). It has been proposed that the rational manipulation of poultry feed formulation can improve pathogen resistance, improve production, and reduce the threat posed by zoonotic pathogens through the food chain (Kogut, 2009; Swaggerty et al., 2019). Intestinal innate immune responses to feed and pathogen challenges are strongly influenced by the gut microbiota (Kogut et al., 2018). The addition of probiotic microorganisms, prebiotics and phytobiotics in feed are approaches by which the gut microbiota of broiler chickens may be influenced (reviewed by Pedroso et al., 2013; Pourabedin and Zhao, 2015; Van Immerseel et al., 2017; Clavijo and Flórez, 2018). It is proposed that these approaches will be most effective when introduced early in life to establish a robust microbiota that benefits production (Rubio, 2019). We have recently reported that the inclusion of the prebiotic GOS in juvenile broiler feed enhances the growth and feed conversion rates of broiler chickens, increases ileal and cecal IL-17A gene expression and brings about changes in the cecal populations of key *Lactobacillus* spp. (Richards et al., 2019b). GOS represent host-indigestible carbohydrates that have been identified as promoting beneficial bacteria in humans and animals, which include *Bifidobacteria*, *Bacteroides*, and *Lactobacillaceae* (Jung et al., 2008; Hughes et al., 2017; Van Bueren et al., 2017; Tian et al., 2019).

In the present study, we have examined whether the impact of a GOS diet on host fitness, immune response and changes in the gut microbiota would support the clearance of *Campylobacter jejuni* in broiler chickens. For this purpose, we fed isocaloric GOS or control diets from hatch to 20 days of age to modulate the intestinal innate immune status and gut microbiota of broiler chickens. Two approaches were taken to determine the role of development on host response. In one experiment birds were challenged at an early stage of development at 6 days old to determine the persistence of *C. jejuni* in the modified gut environment and assess corresponding intestinal chemokine and cytokine gene expression, and the prevailing intestinal microbiota throughout the typical broiler chicken lifespan of 35 days. In a separate experiment birds were challenged at 20 days old, when campylobacters are frequently first detected in commercial flocks, with similar observations made until the trial ended when birds reached 35 days old.

## Materials and Methods

### Trial Design

Two independently performed trials monitored the effect of dietary GOS on development of the gut innate immune responses and the cecal microbiota of broiler chickens challenged with *C. jejuni* HPC5 at either an early stage of development (6 days old) or late stage (20 days old), the age at which birds often become *Campylobacter* positive in commercial production. Birds were randomly assigned to either a group fed a control diet (referred to as *Campylobacter*) or to a group fed a GOS-supplemented diet (referred to as GOS + *Campylobacter*) for the duration of the experiment. In the early 6-day old challenge experiment (referred to as 6-dc), two groups of 35 birds were kept in pens from day of hatch until day 6 when all birds were administered *C. jejuni*, and subsequently independently caged until the end of the study on day 35. Birds were randomly selected (*n* = 7) from each diet group and euthanized prior to sampling intestinal tissues and contents at 8, 15, 22, 28, and 35 days of age (da). For the late 20-day challenge trial (referred to as 20-dc), two groups of 21 birds were similarly housed in pens until 20 days when the birds were administered *C. jejuni* and independently caged until the end of the study at 35 days. Again, randomly selected birds (*n* = 7) from each diet group were euthanized for intestinal sampling at 22, 28, and 35 days. All experimental birds post challenge were maintained in independent housing to prevent the birds sharing intestinal microbiota through coprophagy, which would otherwise confound the experimental design by reducing the number of replicates.

### Experimental Animals

Day-of-hatch male Ross 308 broiler chicks purchased from a local hatchery were randomly assigned on the basis of weight to control or GOS diet groups. Birds were brooded in floor pens on wood shavings until the day of *Campylobacter* challenge. Birds were housed in a controlled environment under strict conditions of biosecurity and kept under controlled light (L:D 12:12) with *ad libitum* access to food and water throughout the study. Temperatures conformed to the Code of Practice for the Housing and Care of Animals Bred, Supplied or Used for Scientific Purposes 2014. Welfare monitoring of the chickens was undertaken two or three times every 24 h post *Campylobacter* challenge. Birds in the control group were sustained on a wheat-based diet provided as starter crumb for 0–10 days, grower pellets for 11–24 days and finisher pellets for 25–35 days. The starter diet contained wheat (59.9% w/w), soya meal (32.5% w/w), soybean oil (3.65% w/w), limestone (0.6% (w/w), calcium phosphate (1.59% w/w), sodium bicarbonate (0.27% w/w), the enzymes phytase and xylanase (dosed according to the manufacturer’s instructions; DSM Nutritional Products Ltd., PO Box 2676 CH-4002 Basel, CH) and a vitamin mix containing NaCl salt, lysine HCl, DL-methionine and threonine. The grower and finisher diets increased the wheat content at the expense of soya meal by 2 and 5% w/w, respectively. The 6-dc prebiotic GOS + *Campylobacter* treatment group had the starter feed supplemented with GOS from 1 to 10 days at 3.37% w/w and then 11–35 days at 1.695% w/w, whilst the 20-dc birds were fed 3.37% w/w GOS throughout the experiment. The GOS was provided as Nutrabiotic^®^ GOS that contains 74% GOS w/w dry matter (Dairy Crest Ltd., Davidstow, Cornwall, United Kingdom). GOS preparations contain a mixture of monosaccharides (glucose and galactose) and oligosaccharides (DP2 – DP8) with the exception of lactose that is a residual component of the production process. The enzymatic synthesis of GOS produces β-(1–3) or β-(1–4) or β-(1–6)-linked galactose residues (1 to 7) with a terminal β-(1–3) or β-(1–4) or β-(1–6)-linked glucose. Isocaloric content adjustments for GOS inclusion were for the starter feed (wheat 54.0% w/w) soya meal (33.9% w/w) and soybean oil (4.88% w/w); for the grower feed (wheat 54.7% w/w) soya meal (32.2% w/w) and soybean oil (6.76% w/w); for the finisher feed (wheat 60.33% w/w) soya meal (26.7% w/w) and soybean oil (6.84% w/w). The feed and paper liners on which the chicks were delivered were found negative for *Salmonella* using standard enrichment procedures. At the time of challenge all birds were administered by oral gavage a dose of 1 × 10^7^ CFU *C. jejuni* HPC5, a well-characterized broiler chicken isolate, suspended in MRD (Oxoid, Thermo Fisher Scientific, Altrincham, United Kingdom) in volumes of 0.1 ml for the 6-dc birds or in 1 ml for the 20-dc birds. For sample collection, birds were euthanized by either exposure to rising CO_2_ gas or parenteral barbiturate overdose followed by cervical dislocation depending on bird mass in accordance with Schedule 1 of the United Kingdom Animals (Scientific Procedures) Act 1986. Ileal tissues were collected from approximately 3 cm distal to Meckel’s diverticulum and cecal tissues isolated from the distal tips of the cecal pouches. Samples of intestinal tissue were immediately frozen in liquid nitrogen for subsequent RNA isolation or preserved in 10% (w/v) neutral buffered formalin (Thermo Fisher Scientific) for histological assessment. Cecal contents were collected and used either to enumerate *Campylobacter* or for isolation of total genomic DNA extraction.

### Performance and Growth Rate

Live weights and all feed consumed were recorded for all birds at regular intervals from the start of the experiment until the end at 35 days. FCR were calculated as a ratio of feed consumed to the live weight of the birds. Bird growth rates were compared for each of the birds that remained at the end of the 35 days rearing period that collectively represent all the birds for which repeated measurements of the mass were recorded throughout the broiler chicken lifespan.

### Bacterial Enumeration

Approximately 1 g of digesta was aseptically collected from both ceca and combined in pre-weighed universal containers before a 10% w/v suspension was prepared in MRD. *Campylobacter* were enumerated in triplicate from decimal dilutions prepared in MRD using a modification of the Miles and Misra technique (Miles et al., 1938). For each triplicate dilution set, five aliquots were dispensed onto CCDA agar (PO0119; Oxoid) prepared with the addition of agar to 2% (to prevent swarming) and with addition of CCDA Selective Supplement SR0155 (Oxoid). Plates were incubated at 42°C in a microaerobic atmosphere (2% H_2_, 5% CO_2_, 5% O_2_, and 88% N_2_ v/v) for 48 h (Don Whitley Scientific modified atmospheric cabinet, Shipley, United Kingdom).

### Histology

Tissue samples fixed in a 10% formalin solution were dehydrated through a series of alcohol solutions, cleared in xylene, and embedded in paraffin wax (Microtechnical Services Ltd., Exeter, United Kingdom). Sections (3 to 5 μm thick) were prepared and stained with modified hematoxylin and eosin (H&E). After staining, the slides were scanned by NanoZoomer Digital Pathology System (Hamamatsu, Welwyn Garden City, United Kingdom). Villus height and crypt depth were recorded from operator blinded measurements collected using the NanoZoomer Digital Pathology Image Program (Hamamatsu) from histology stained slides scanned at 40× resolution for each tissue sample. Villus height was determined from the tip of the villus to the crypt opening and the associated crypt depth was measured from the base of the crypt to the level of the crypt opening. The ratios of villus height to relative crypt depth (v/c ratio) were calculated from these measurements. Dimensions for 10 well-oriented villi per tissue sample of 3 or 4 birds per diet group at each sampling time were analyzed.

### RNA Isolation and RT-qPCR of the Cytokines and Chemokines

Total RNAs were isolated from ceca and ileum tissue biopsies using NucleoSpin RNA purification kit (Macherey-Nagel, GmbH & co. KG, Düren; DE) according to the manufacturer’s protocol with the following modifications. Tissue samples were homogenized with the kit Lysis buffer and 2.8 mm ceramic beads (MO BIO Laboratories Inc., Carlsbad, United States) using TissueLyser II (Qiagen, Hilden, Germany). Subsequently total RNAs were extracted as described in the protocol with a DNaseI treatment step as per the manufacturer’s instructions. Purified RNAs were eluted in nuclease free water, validated for quality and quantity using UV spectrophotometry (Nanodrop ND-1000, Labtech International Ltd., Uckfield, United Kingdom), and stored long term at −80°C. RNAs with OD260/280 ratio between 1.9 and 2.1 were deemed high quality, the ratios were found with a mean of 2.12 ± 0.01. Reverse Transcription was performed with 1 μg of RNA, SuperScript II (Invitrogen Life Technologies, Carlsbad, CA, United States) and random hexamers as described previously (Connerton et al., 2018). Quantitative PCR reaction was performed with cDNA template derived from 4 ng of total RNA in triplicate using SYBR Green Master mix (Applied Biosystems, Thermo Fisher Scientific). The RNA level of expression was determined by qPCR using the Roche Diagnostics LightCycler 480 (Hoffmann La Roche AG, CH). The primers sequence for GAPDH, INF-γ, IL-1β, IL-6, IL-10, IL-17A, IL-17F, ChCXCLi1, and ChCXCLi2 (Table 1) were previously described (Kaiser et al., 2003; Nang et al., 2011; Rasoli et al., 2015; Reid et al., 2016). Cytokines and chemokines transcripts fold change (FC) were calculated according to the manufacturer using the 2^–Δ^ ^Δ^ ^*Cp*^ method (Livak and Schmittgen, 2001). Averages of the triplicate Ct values were analyzed with the target genes of interest (GOI) values normalized to those of the housekeeping gene Glyceraldehyde 3-phosphate dehydrogenase (GAPDH).

**TABLE 1:** Primer sequences for the gene expression determined by qPCR.

**Target gene**	**Primer sequence (5′-3′)**	**Product size (bp)**	**NCBI Accession number**	**References**
GAPDH	F: GACGTGCAGCAGGAACACTA R: TCTCCATGGTGGTGA AGACA	343	NM_204305.1	Nang et al. (2011)
INF-γ	F: TGAGCCAGATTGTTTCGATG R: CTTGGCCAGGTCCATGATA	152	NM_205149.1	Nang et al. (2011)
IL-1β	F: GGATTCTGAGCACACCACAGT R: TCTGGTTGATGTCGAAGATGTC	272	NM_204524.1	Nang et al. (2011)
IL-10	F: GCTGCGCTTCTACACAGATG R: TCCCGTTCTCATCCATCTTC	203	NM_001004414.2	Nang et al. (2011)
IL-6	F: GCTCGCCGGCTTCGA R: GGTAGGTCTGAAAGGCGAACAG	71	NM_204628.1	Kaiser et al. (2003)
IL-17A	F: CATGGGATTACAGGATCGATGA R: GCGGCACTGGGCATCA	68	NM_204460.1	Reid et al. (2016)
IL-17F	F: TGACCCTGCCTCTAGGATGATC R: GGGTCCTCATCGAGCCTGTA	78	XM_426223.5	Reid et al. (2016)
ChCXCLi-1	F: CCGATGCCAGTGCATAGAG R: CCTTGTCCAGAATTGCCTTG	191	NM_205018.1	Rasoli et al. (2015)
ChCXCLi-2	F: CCTGGTTTCAGCTGCTCTGT R: GCGTCAGCTTCACATCTTGA	128	NM_205498.1	Rasoli et al. (2015)

### Microbiota Analysis

DNA was isolated from cecal content using the MoBio PowerSoil kit (now QIAGEN Ltd., Manchester, United Kingdom) according to the manufacturer’s instructions. The V4 regions of the bacterial 16S rRNA genes were PCR amplified using the primers 515f (5′ GTGCCAGCMGCCGCGGTAA 3′) and 806r (5′ GGACTACHVGGGTWTCTAAT 3′) (Caporaso et al., 2011). Amplicons were then sequenced on the Illumina MiSeq platform (Illumina Inc., San Diego, United States) using 2 × 250 bp cycles (reagent kit V2). The 16S rRNA gene sequences were quality filtered and clustered into OTUs in Mothur (Schloss et al., 2009) using the Schloss lab. MiSeq SOP^1^, accessed 2018-10-05; Kozich et al., 2013). Batch files of Mothur commands used in this study are available at https://github.com/PJRichards/lafontaine_campy_gos. Raw sequences for 16S rDNA data originally reported in this article are deposited in the NCBI database within BioProject PRJNA380214 under SRA study SRP133552. Comparative 16S rDNA data from mock-challenged birds reproduced in this study was downloaded from NCBI PRJNA380214 (the FTP code is available from GitHub repository as described below). Post-processing rarefaction curves were plotted to assess sampling effort (Supplementary Figure S1).

### Data and Statistical Analysis

All figures were drawn and unless otherwise stated all tests for statistical significance were performed using R 3.6.1 (R Core Team, 2019) in RStudio 1.2.1 (RStudio Team, 2015). All R scripts have been made available here: https://github.com/PJRichards/lafontaine_campy_gos. Histology measurements for each diet regimen of age-matched birds were compared using ANOVA.

### Zootechnical Data

Bird growth rates were compared by determining rate of growth from 15 days for each of the birds that remained at the end of the trial at 35 days, i.e., birds for which repeated measurements of the mass were recorded throughout the growing period (*Campylobacter* treatment group, *n* = 7; GOS + *Campylobacter* treatment, *n* = 8). Growth rate was determined for individual birds for the period of linear growth post-challenge (6-dc birds between 15 and 35 days; 20-dc birds between 22 and 35 days). Growth rates (g/day) were compared between cohorts using Student’s *t* test. Further comparison was made between the mass of age-matched birds using Student’s *t* test. *C. jejuni* viable counts were log_10_-transformed and tested for significance using Student’s *t* test.

### Microbiota – 16S rRNA Gene Sequence Data

Comparisons were made of α-diversity metrics (Shannon diversity and inverse Simpson’s indices) generated in mothur. For the four treatment groups in 6-dc birds at 8 days (2 dpi) differences in α-diversity were tested for using ANOVA with Tukey multiple comparison of means test (*p* was adjusted for multiple comparisons). For subsequent comparisons of α-diversity at 15, 22, 28 and 35 days between two treatment groups only (*Campylobacter* and GOS + *Campylobacter*) Student’s *t* test was used to test for significance. Correspondingly, comparisons of Chao richness between the four treatment groups in 6-dc birds at 8 days (2 dpi) were made using a Kruskal-Wallis test with Benjamini-Hochberg FDR correction as were subsequent comparisons at 15, 22, 28, and 35 days. Note that a randomly selected community was deleted from the GOS + *Campylobacter* treatment group at 15 days and 35 (birds 2 and 6, respectively) to even group size and allow unbiased comparisons. For 20-dc birds, comparisons of α-diversity were made using Student’s *t* test and comparisons of Chao richness were made using a Kruskal-Wallis test. Differences in bacterial composition were tested for by modeling compositional population data in terms of a Dirichlet distribution and using a likelihood ratio test in DirtyGenes (Shaw et al., 2019). Differential OTUs were identified with LEfSE in mothur (Schloss et al., 2009; Segata et al., 2011).

### Intestinal Cytokine and Chemokine Transcription

Host cytokine and chemokine transcript levels were assessed by RT-qPCR of transcribed RNA isolated from ileal and cecal tissue sections. Cytokine and chemokine normalized expression was determined for each sample as 2^–Δ^ ^*Cp*^ with ΔCp = Cp of GoI – Cp of housekeeping gene (GAPDH). The relative gene expression between birds fed a control (*Campylobacter*) or a GOS diet (GOS + *Campylobacter*), results were determined as a group mean FC, which was calculated from 2^–Δ^ ^Δ^ ^*Cp*^ with ΔΔCp = ΔCp (GOS diet) - ΔCp (average ΔCp of control). Differences between treatment groups were assessed using Wilcoxon rank sum tests with Benjamini-Hochberg FDR correction.

## Results

### Dietary Galacto-Oligosaccharide Improved the Growth Performance of *Campylobacter jejuni*-Colonized Broiler Chickens

**FIGURE 1:**
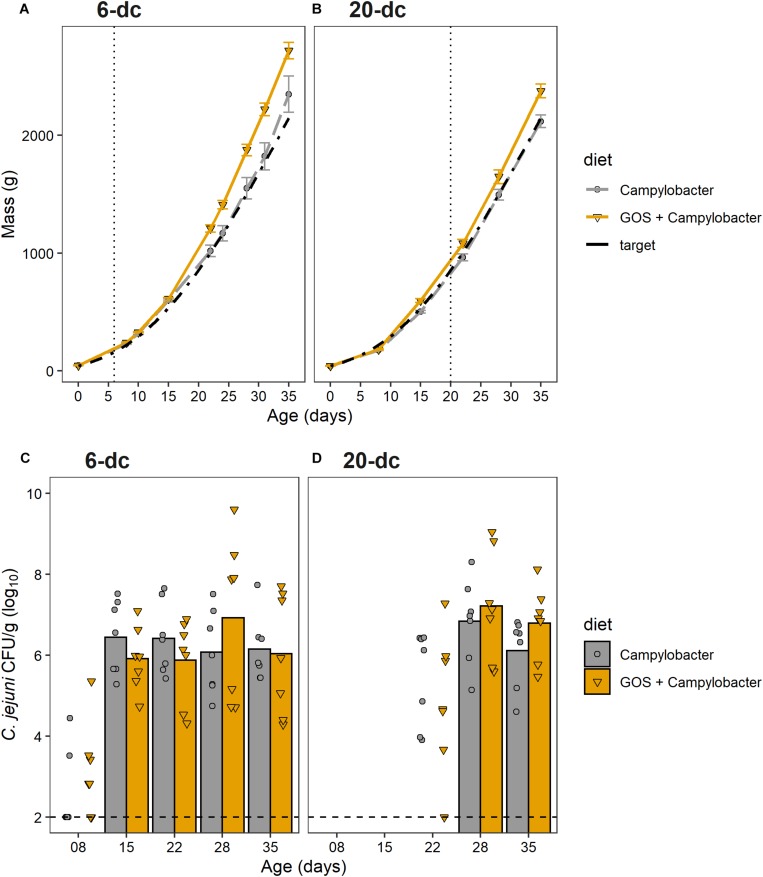
GOS improves growth performance of broiler chickens, but does not affect *C. jejuni* colonization of the ceca. **(A,B)** Chicken live total mass from day of hatch to 35 days for 6-dc birds **(A)** and 20-dc birds **(B)**. The plots show the performances of the experimental treatments GOS + *Campylobacter* (GOS diet) *Campylobacter* (control diet) and the Ross 308 performance objective as the Target (Aviagen Performance Objectives, 2014). The dashed line indicates age at challenge. **(C,D)** Viable counts of *Campylobacter* recovered from the cecal content of 6-dc birds **(C)** and 20-dc birds **(D)**. Data markers indicate *Campylobacter* CFU isolated from individual birds. Bars indicate mean *Campylobacter* CFU, excluding cohorts where *Campylobacter* levels were below the limit of detection. There are no significant differences in the *Campylobacter* counts for the GOS diet compared to control diet post colonization (*p* > 0.05). The dashed line indicates minimum level of detection.

The aim of this research was to determine whether GOS could still act as a prebiotic and confer a growth performance advantages to broiler chickens colonized when colonized by *C. jejuni*. Chickens fed a GOS diet performed better than those fed the calorie-matched control diet (Figures 1A,B). Differences in the body weights were evident from 22 days for the 6-dc birds (*p* ≤ 0.029) until slaughter at 35 days (mean body weights *Campylobacter* treatment = 2276 g, GOS + *Campylobacter* treatment = 2722; *p* = 0.029) (Figure 1A). Differences in the weights of the 20-da challenged birds were observed at 35 days (mean body weights *Campylobacter* = 2055 g, GOS + *Campylobacter* = 2341; *p* = 0.004) (Figure 1B). The growth rates of the 6-da challenged birds fed GOS increased in the period 15–35 days compared to the challenged chickens fed a control diet (control = mean 83.6 g/day, GOS = mean 105.1 g/day; *p* = 0.0233), and similarly for the 20-da challenged birds for the period 22–35 days (control = mean 85.3 g/day, GOS = mean 97.6 g/day; *p* = 0.007).

The cumulative FCR up to 35 days for 6-dc birds fed control diet (*n* = 7) was 1.45 and for the GOS diet (*n* = 8) was 1.42 while the FCR for 20-dc birds fed control diet (*n* = 7) was 1.56 and for GOS diet (*n* = 7) was 1.58 (data not shown). The contemporary breed performance objectives for male Ross 308 were body weight 2,283 g and FCR of 1.54 at 35 days (Aviagen Performance Objectives, 2014).

### Dietary Galacto-Oligosaccharide Inclusion Did Not Prevent *Campylobacter jejuni* Colonization of Broiler Chickens

To assess the impact of dietary GOS on *C. jejuni* colonization we sacrificed birds over the rearing period to determine the cecal viable *Campylobacter* counts (*n* = 7). All birds were culture-negative for *Campylobacter* spp. until oral gavage with *C. jejuni* HPC5 at 6 days for the early-challenge cohort (6-dc) or 20 days for the late-challenge birds (20-dc). Birds challenged at 6 days (Figure 1C) showed incomplete colonization at 2 dpi (2/7 and 3/7 for the control and GOS cohorts, respectively), but the treatment groups all showed complete colonization with *C. jejuni* at 9 dpi. Mean colonization levels of 6.4 log_10_ CFU/g for the control diet and 5.9 log_10_ CFU/g for the GOS diet were recorded (15 days). The birds then remained colonized thereafter to the end of the 35 days rearing period with no significant differences between the colonization levels of the dietary groups at any time (Figure 1C). The 20-dc birds (Figure 1D) were all found colonized at 8 dpi with mean colonization levels of 6.8 log_10_ CFU/g for the control and 7.2 log_10_ CFU/g for the GOS diet (28 days). Viable counts of *Campylobacter* in cecal content remained high at the end of the rearing period with no significant differences between the diets at any time, independent of the age of challenge.

### Intestinal Villus and Crypt Metrics Were Affected by Dietary Galacto-Oligosaccharide Post-infection With *Campylobacter jejuni*

Villus and crypt metrics were determined from 10 well-oriented villi for 3 to 4 birds from each group in a blind assessment of formalin-fixed H&E-stained ileum sections. Measurement comparisons of the GOS and control diet groups for 6-dc birds showed greater villus length at 8 (2 dpi; *p* = 0.04) and 15 days (9 dpi; *p* = 0.002) for the *C. jejuni* colonized GOS-fed chickens compared to the *C. jejuni* colonized birds on control feed (Table 2). By 22 days the villus lengths of the 6-dc treatment groups were not significantly different and remained so until the end of the trial at 35 days. Comparison of the crypt depth measurements demonstrated that the GOS-fed birds at 15 days (*p* = 0.005) had significantly deeper crypts than the birds on the control diet. Differences in the villus and crypt measurements affected differences in the villus to crypt ratios at 8 days (*p* = 0.02) and 15 days (*p* = 0.04) with the GOS-fed birds exhibiting greater ratios. For the 20-dc cohorts the GOS-fed birds also exhibited significant increases in villus height compared to the control diet after *C. jejuni* colonization at 22 days (2 dpi; *p* = 0.04) and 28 days (8 dpi; *p* = 0.05). A significant increase in the crypt depth for the GOS-fed birds over the birds on the control diet was also recorded at 22 days (*p* = 0.05), but not thereafter. These differences did not result in significant differences in the villus to crypt ratios for the 20-dc experiment.

**TABLE 2:** Ileal histomorphometry.

	**Histology measurements**									
	
	**8 days**	**SD**	**15 days**	**SD**	**22 days**	**SD**	**28 days**	**SD**	**35 days**	**SD**
**Villus length (μm)**										
Campy (6-dc)	550	74	560	64	803	33	940	71	934	32
Campy + GOS (6-dc)	671	15	796	58	824	61	814	63	888	40
Campy (20-dc)	–		–		636	56	746	88	954	98
Campy + GOS (20-dc)	–		–		766	82	922	114	1092	124
*p-value* (6-dc)	0.04		0.002		0.56		0.09		0.31	
*p-value* (20-dc)	–		–		0.04		0.05		0.13	
**Crypt depth (μm)**										
Campy (6-dc)	117	12	111	7	125	8	128	5	121	8
Campy + GOS (6-dc)	110	8	133	4	128	5	132	8	122	11
Campy (20-dc)	–		–		82	16	92	24	104	22
Campy + GOS (20-dc)	–		–		106	12	102	38	128	44
*p-value* (6-dc)	0.32		0.005		0.65		0.49		0.69	
*p-value* (20-dc)	–		–		0.05		0.67		0.37	
**Villus length/Crypt depth ratio (v/c)**										
Campy (6-dc)	4.70	0.84	5.05	0.60	6.42	0.17	7.34	0.63	7.72	0.82
Campy + GOS (6-dc)	6.10	0.38	5.98	0.39	6.44	0.31	6.17	0.59	7.28	0.27
Campy (20-dc)	–	–	–	–	7.76	0.80	8.11	0.74	9.17	0.91
Campy + GOS (20-dc)	–	–	–	–	7.23	0.91	9.04	0.82	8.53	0.72
*p-value* (6-dc)	0.02		0.04		0.83		0.08		0.35	
*p-value* (20-dc)	–		–		0.42		0.14		0.31	

*Villus length, crypt depth and ratio villus length/crypt ratios presented as mean values (±standard deviation) of the measurement of 10 well-orientated villi for 3 to 4 birds per group. Corresponding probabilities (*p*) were calculated using ANOVA tests, differences were considered significant at *p* < 0.05.*

### Dietary Galacto-Oligosaccharide Modulates IL-17 Transcription Post *Campylobacter* Colonization

Immuno-modulatory effects of dietary GOS on the innate immune responses of intestinal tissues from *C. jejuni*-colonized broilers were assessed. RNAs were extracted from biopsies collected from ileal and cecal tissues to enable RT-PCR quantification of cytokine and chemokine gene transcripts representing the major inflammatory pathways of chickens. Cytokines IL-17A, IL-17F, IL-6, IL-1β and chemokines ChCXCLi-1, ChCXCLi-2 (also known as ChIL-8) have previously been described as markers of the Th17 pathway (Reid et al., 2016). IFN-γ is related to the Th1 pathway and the anti-inflammatory cytokine IL-10 is largely expressed from regulatory T cells (Treg) in chickens to control the inflammatory effects of the Th cell responses.

**FIGURE 2:**
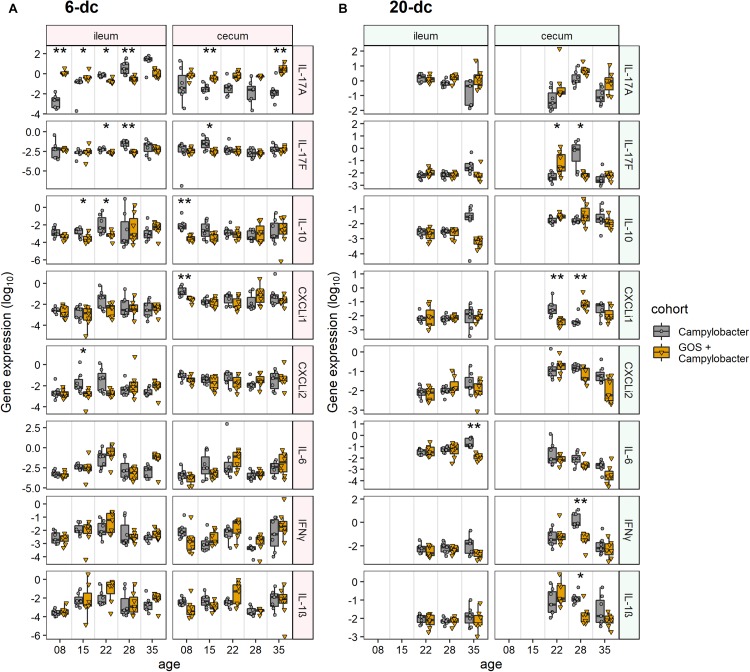
Modulation of cecal and ileal innate immune responses to dietary GOS in *Campylobacter*-challenged broiler chickens. The figures report cytokine and chemokine normalized gene expression for 6-dc birds **(A)** and 20-dc birds **(B)** fed a control diet (*Campylobacter* treatment) or a GOS diet (GOS + *Campylobacter* treatment) recorded as log_10_ of the ratio for gene of interest/GAPDH from qPCR data of individual birds. Corresponding probabilities (*p-adj*) were calculated using non-parametric Wilcoxon rank sum tests with Benjamini-Hochberg FDR correction, where differences at *p-adj* < 0.05 were considered significant and are summarized in the plot using asterisks (^∗^*p-adj* ≤ 0.05; ^∗∗^*p*-*adj* ≤ 0.01).

The innate immune response of ileal tissues for the early challenged birds (6-dc) was characterized by modulation in the expression of IL-17A in *C. jejuni-*colonized birds on control feed compared to *C. jejuni-*colonized GOS-fed birds (Figure 2A). Following *Campylobacter* challenge IL-17A transcript levels were far greater in the GOS-fed birds (884-fold difference) than those on the control diet at 8 days (2 dpi; *p-adj* = 0.005). By 15 days the difference had declined to 12-fold due to an increase in IL-17A in the *C. jejuni-*colonized birds on the control diet (9 dpi; *p-adj* = 0.02). The rise in IL-17A continued in the birds on the control diet until 35 days such that a significant difference was recorded in favor of the control diet birds from 22 days (16 dpi; *p-adj* = 0.02), whilst the IL-17A levels were maintained in the GOS-fed birds throughout the time course. The pro-inflammatory cytokine IL-17F exhibited a significant increase in expression at 22 days (16 dpi; *p-adj* = 0.03) and 28 days (22 dpi; *p-adj* = 0.005) in the control birds. Over the transition period, the regulatory cytokine IL-10 expression was significantly greater at 15 days (9 dpi; *p-adj* = 0.05) and 22 days (16 dpi; *p-adj* = 0.03) in the *C. jejuni-*colonized birds on control diet compared to the GOS diet (GOS + *Campylobacter* treatment). For the late challenged birds (20-dc; Figure 2B), IL-6 was recorded as significantly greater for the *C. jejuni-*colonized birds on the control diet at 35 days (29 dpi; *p-adj* = 0.005), largely owing to a fall in the IL-6 levels of the birds on the GOS diet (GOS + *Campylobacter* treatment).

In cecal tissues IL-17A expression was also maintained in the GOS-fed birds post *C. jejuni* colonization. In the early challenge experiment (6-dc) IL-17A expression levels declined in the birds fed the control diet throughout the time course with significant differences recorded at 15 days (9 dpi; *p-adj* = 0.005) and 35 days (288-fold at 29 dpi; *p-adj* = 0.01) compared to the birds on the GOS containing diet (Figure 2A). Pro-inflammatory IL-17F exhibited a significant increase at 15 days in the *C. jejuni-*colonized birds fed the control diet compared to the birds on the GOS diet (9 dpi; *p-adj* = 0.04). This was preceded by differential increases at 8 days in IL-10 (2 dpi; *p-adj* = 0.002) and ChCXCLi-1 (2 dpi; *p-adj* = 0.002). The late challenged birds (20-dc) featured a significant switch in the expression of IL-17F from low to high for the *C. jejuni-*colonized birds on the control diet between 22da (2 dpi; *p-adj* = 0.04) and 28 days (8 dpi; *p-adj* = 0.02), and conversely the birds on the GOS diet exhibited a high to low change in gene expression over the period (treatment GOS + *Campylobacter*). Increases in IL-17F were accompanied by significant increases in IFN-γ (8 dpi; *p-adj* = 0.005) and IL-1β (8 dpi; *p-adj* = 0.02) at 22da for the *C. jejuni-*colonized birds on the control diet. Transcription of the chemokine ChCXCLi-1 was observed to exhibit the opposite trend to IL-17F over the 22 days (2 dpi; *p-adj* = 0.005) to 28 days (8 dpi; *p-adj* = 0.005) transition, with a fall in the mean expression value for the *C. jejuni-*colonized birds on the control diet compared to an increase in the *C. jejuni-*colonized GOS-fed birds (treatment GOS + *Campylobacter* in Figure 2B).

### Galacto-Oligosaccharide -Induced Microbiota Diversity Shifts in *Campylobacter*-Challenged Birds

**FIGURE 3:**
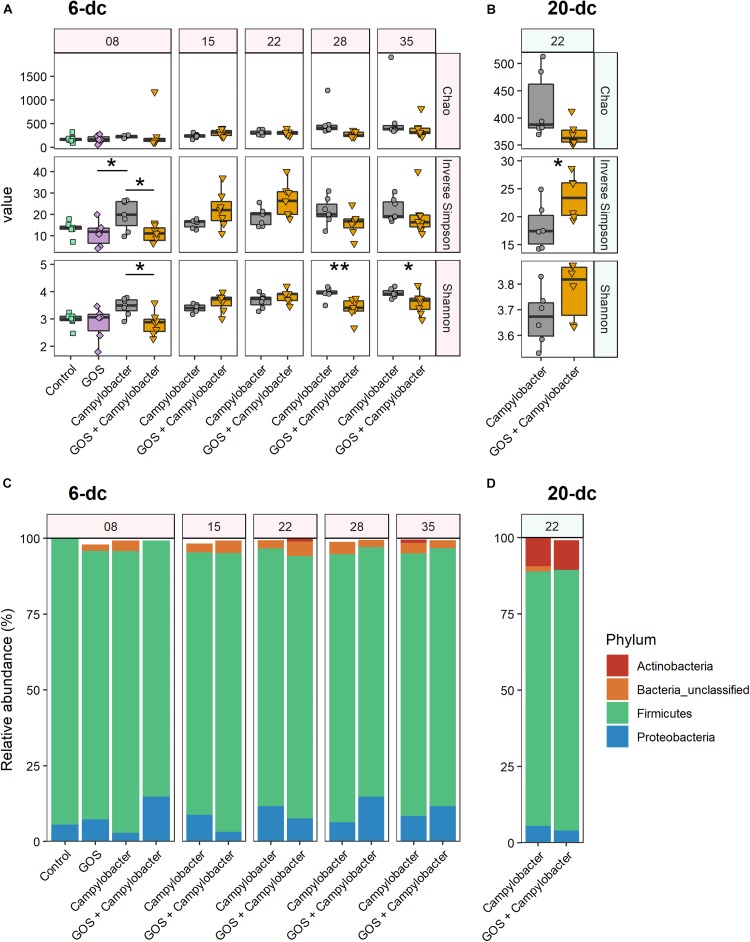
Dietary GOS promotes differential shifts in the cecal microbiota post *Campylobacter* challenge. **(A,B)** Cecal α-diversity for 6-dc birds **(A)** and 20-dc birds **(B)** described as Chao richness, Inverse Simpson diversity and Shannon diversity (as indicated by panels on right hand side of the figure). The age of the birds are in days as indicated by the numerals in the strip at the top of the figure. Significant differences between groups are indicated by asterisks (^∗^*p* ≤ 0.05; ^∗∗^*p* ≤ 0.01). **(C,D)** OTU relative abundance for 6-dc birds **(C)** and 20-dc birds **(D)** summarized at Phyla level.

At 2 dpi (8 days) the α-diversity of the cecal microbiota of 6-dc birds was lower in GOS + *Campylobacter* treated birds than the *C. jejuni-*colonized birds on the control diet (treatment *Campylobacter*), as indicated by lower Shannon entropy (*p* = 0.046) and inverse Simpson index (*p* = 0.022; Figure 3A). This may be attributed to the dietary GOS as the inverse Simpson’s index of mock-challenged birds on a GOS diet was also lower than that of the *Campylobacter* treatment birds on the control diet (*p* = 0.032), with the difference observed in the corresponding Shannon entropy values approaching the significance threshold (*p* = 0.058). Shannon entropy was also lower in GOS + *Campylobacter* treatment birds at 28 days (22 dpi; *p* = 0.009) and 35 days (29 dpi; *p* = 0.038) as presented in Figure 3A. There were no other observed differences in α-diversity or species richness (Chao) in 6-dc birds. In the 20-dc experiment at 22 days (2 dpi) the inverse Simpson index of the cecal microbiota of the GOS + *Campylobacter* treatment birds was significantly greater than *Campylobacter* treatment birds (*p* = 0.0469; Figure 3B). The responses recorded for the inverse Simpson index at 2 dpi for the 6-dc and 20-dc experiments show opposite trends, however, a similar trend can be noted in the higher α-diversity of 6-dc GOS + *Campylobacter* treatment birds at the same age (22 days), although these changes did not reach significance. The modulation in α-diversity at this age could be attributable to changes in host development or changes in diet formulation of grower to finisher related to husbandry.

Comparison of the phylum composition of 6-dc birds at 8 days (2 dpi) indicates that the cecal microbiota of *Campylobacter* treatment birds was significantly different from the microbiota of the GOS + *Campylobacter* treatment birds (*p* = 0.0003) (Figure 3C). Comparison of the microbiota of GOS + *Campylobacter* treatment birds with the cecal microbiota of age-matched mock-challenged birds on the GOS diet alone at 8 days (2 dpi) did not reveal any phyla-level differences (*p* = 0.1613), whilst mock-challenged birds on either control or GOS diets also had different phyla compositions (*p* = 0.0096). These data suggest that the differences in microbiota ecology are linked to dietary GOS and not *Campylobacter*-colonization *per se*. No difference in phylum-composition was determined in 6-dc birds at 15 days (9 dpi; *p* = 0.301), 22 days (16 dpi; *p* = 0.69) or 35 days (*p* = 0.055). However, the composition of the cecal microbiota of birds from the GOS + *Campylobacter* and the *Campylobacter* treatment groups were different at 28 days (22 dpi; *p* = 0.0004), which likely corresponds with the reverse in α-diversity first observed at this age. No differences were determined in the phylum-composition of 20-dc birds at 2 dpi (*p* = 0.742) (Figure 3D).

At OTU level 16S rRNA gene sequencing reads were clustered at 97% similarity, which serves as a proxy for species-level distinction. The 6-dc birds at 8 days (2 dpi) show a key differential OTU between *Campylobacter*-colonized birds on the control diet (*Campylobacter* treatment) versus *C. jejuni-*colonized birds on the GOS diet (GOS + *Campylobacter* treatment) had strong sequence similarity to *Escherichia coli* (OTU0001; 99.21%, Supplementary Figure S2A). OTU0001 did not discriminate the microbiota of the *Campylobacter*-challenged birds on the control diet (*Campylobacter* treatment) from the microbiota of mock-challenged birds on the GOS diet (Supplementary Figure S2C). In addition, previous analysis of the mock-challenged controls by our laboratory indicated that *Lactobacillus johnsonii* outcompetes *L. crispatus* in GOS-fed birds (Richards et al., 2019b). In the analysis presented here OTU0002 has strong sequence similarity with the *L. crispatus* strain (97.21%) and OTU0017 has a strong sequence similarity with *L. johnsoniii* (98.03%). In 6-dc birds at 8 days (2 dpi) both OTU0002 (*L. crispatus*) and OTU0017 (*L. johnsoniii*) are associated with dietary GOS (Supplementary Figure S2B). However, *L. johnsoniii* (OTU0017) continues to be associated with dietary GOS at: 15 days (9 dpi), 22 days (16 dpi) and 28 days (22 dpi). Whereas, *L. crispatus* (OTU0002) is later associated with the control diet at 15 days (9 dpi) and 35 days (29 dpi).

## Discussion

**FIGURE 4:**
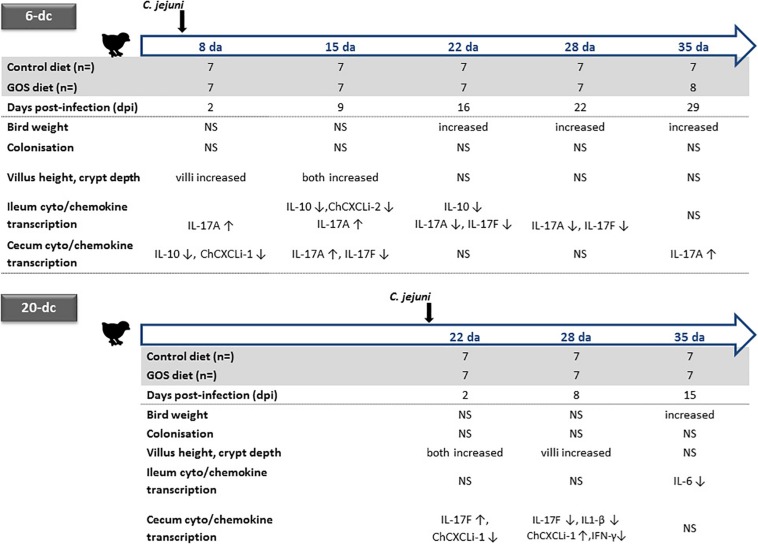
Summary of age dependent differences for dietary GOS in *C. jejuni* colonized broiler chickens. Significant changes in the live weight, cecal *C. jejuni* colonization levels, ileal villus and crypt metrics are indicated for the GOS-fed birds compared to the birds on the control diet colonized at either 6 or 20 days of age (da) with *C. jejuni*. Relative changes in the transcription of ileal and cecal cytokines and chemokines for GOS-fed bird compared to controls are indicated for age matched birds by up arrows (↑) for increases and down arrows for decreases in cytokine/chemokine expression (↓). NS indicates no significant differences between the birds on the GOS or control diet.

Concerns are growing regarding the over use of antimicrobials in animal production, and any concomitant increase in risk of antimicrobial resistance transferring to humans (Landers et al., 2012). These concerns have brought about calls for restricting antibiotic use in food producing animals (Tang et al., 2017). Although the European Union banned antimicrobial growth promoters since 2006, the practice continues in many countries and has prompted calls for a worldwide ban, particularly in the poultry and pig industries. It is becoming increasingly evident that improved on farm-performance at the expense of intestinal health and the zoonotic dissemination of antimicrobial resistance cannot continue (Awad et al., 2015, 2017; Smith et al., 2016). Under increasing economic pressure, the poultry industry is pursuing effective alternative methods to promote bird growth whilst controlling farm sources of antimicrobial resistance and zoonotic disease. Prebiotic feed additives such as GOS, a by-product of the dairy industry, have revealed potential growth-promoting effects in piglets (Alizadeh et al., 2016) and chickens (Richards et al., 2019b), whilst fructo-ologosaccharide (FOS) have been reported to reduce *Salmonella* colonization of chicks (Fukata et al., 1999). Here, we assess the impact of a GOS prebiotic on chickens colonized by *C. jejuni* at either 6 or 20 days with respect to their zootechnical performance, gut architecture and differences in intestinal cytokine and chemokine transcription (the experimental designs and corresponding data are summarized in Figure 4).

Two independent trials show significant differences in live bodyweights of *C. jejuni*-challenged birds, the birds fed GOS additive were significantly heavier at the typical market age of 35 days compared to a calorie-match control diet. GOS supplementation improved the growth rate performance of *Campylobacter*-colonized broiler chickens irrespective of the timing of the challenge with *C. jejuni* at either 6 days or 20 days of age. However, dietary GOS inclusion did not prevent or reduce *C. jejuni* HPC5 colonization of broiler chickens GIT within the 35 days lifespan of the birds. *C. jejuni* HPC5 is a broiler chicken isolate that has been used routinely and reliably to colonize the intestinal tract of broiler chickens (Loc Carrillo et al., 2005; Scott et al., 2007; Connerton et al., 2018). In recent years several studies have employed 16S rRNA gene sequences to comprehensively document changes in the cecal microbiota that accompany *Campylobacter*-colonization of broilers. These studies have noted differences in the relative abundance of Bifidobacterium, Lactobacillaceae, Clostridium cluster XIVa and Mollicutes, with transient age related shifts in specific members of the Lachnospiraceae and Ruminococcaceae (Thibodeau et al., 2015, 2017; Connerton et al., 2018; Richards et al., 2019a). As an extension of the outputs from these studies it was recognized that transitions in the cecal microbiota were evident between 14 and 18 days that coincide with the reduced availability of maternal antibodies, and represent a window of opportunity for the entry for bacteria to bloom and new intestinal microbes to become established that can affect changes in gut health (Awad et al., 2016; Connerton et al., 2018; Ijaz et al., 2018). Early prebiotic diets offer the prospect of achieving a stable microbiota that can resist opportunist expansion or colonists. Prebiotic oligosaccharides have previously been reported to reduce the cecal *C. jejuni* colonization loads of broiler chickens, for example the use of a chicory fructan additive for 42 days in male Ross 308 birds (Yusrizal and Chen, 2003), or as the use of mannan-oligosaccharide (MOS) with male Cobb 500 birds at 34 days (Baurhoo et al., 2009). It is conceivable that improved broiler breeding programs and optimized diets will produce heavier birds faster, which will require fast-resolving methods that reduce or displace unwelcome gut bacteria.

Intestinal histomorphometric parameters are considered indicators of gut health whereby a healthy ileal mucosa should display long villi with high villus/crypt ratios. A transient increase in villus length and crypt depth was observed over the first 2 weeks post-challenge for birds sustained on the GOS diet irrespective of the timing of *Campylobacter* challenge. Increases in the absorption surface provide a favorable environment for nutrient uptake leading to efficient feed utilization. Several studies have shown the beneficial effects of dietary additives on Ross 308 broiler chicken villus architecture and body weight gain during heat stress challenges such as prebiotic supplements (Silva et al., 2010), alpha-lipoic acid additive (Yoo et al., 2016), or probiotic mixtures (Song et al., 2014). Similarly, the data presented here suggests that nutrient absorption competence associated with the development of villi in the small intestine of birds fed the GOS diet, leads to an increase in body weight despite gut colonization by *C. jejuni*.

Intestinal mucosa constitutes a physical and immunological protective barrier for the integrity of the intestinal tract to prevent infection by pathogens and maintain an environment that can sustain a healthy and productive microbiota. However, the composition of the gut microbiota is under surveillance of the mucosal innate and adaptive immune systems (Kraehenbuhl and Neutra, 1992). Numerous immune cell populations such as regulatory T-cells (Treg), Th17 cells, IgA-secreting plasma cells, natural killer cells (NK), macrophages, dentritic cells (DCs), innate lymphoid cells (ILCs) contribute to host defense against infection with pathogenic microbes (Honda and Littman, 2012). Members of the IL-17 family of cytokines, IL-17A and IL-17F are produced by a subset of CD4 + T cells named Th17, and have more recently been associated in the gut with dendritic cells and group 3 innate lymphoid cells (ILC3) (Li S. et al., 2018). While they beneficially mediate resistance to extracellular bacterial and fungal infection via enhanced mucosal production of mucus and antimicrobial peptides, IL-17A and IL-17F are also involved in several autoimmune disorders (Bettelli et al., 2007; Yang et al., 2008; Ishigame et al., 2009).

Here, we demonstrate dietary GOS inclusion can maintain transcript levels of IL-17A and suppress IL-17F post colonization with *C. jejuni*. Previously we have shown *C. jejuni* challenge at 6 days triggered a transient increases in IL-17A and IL-17F in broiler chickens at 15 days (9 dpi) compared with non-colonized birds (Connerton et al., 2018), and more recently that dietary GOS increases the expression of IL-17A in juvenile birds up to 15 days (Richards et al., 2019b). In mice IL-17A expression has been proposed to benefit intestinal barrier function and IL-17F to weaken intestinal integrity, since IL-17A inhibition exacerbates induced colitis (Maxwell et al., 2015) and IL-17F suppression is protective (Tang et al., 2018). Evidence suggests the modulation of IL-17A is associated with the regulation of the tight junction formation and regulation of the mucosal barrier via activation of the ERK MAPK pathway (Awane et al., 1999; Cario et al., 2000; Kinugasa et al., 2000). *C. jejuni* colonization of the chicken intestine appears to result in the expression of both the IL-17 subtypes with opposing effects mediated at different times. The dominant response may in part explain why there are differences in the impact of *C. jejuni* colonization reported for different bacterial types on various broiler chicken breeds (Humphrey et al., 2014, 2015). An increase of IL-17A in response to dietary GOS in the presence or absence of *C. jejuni* colonization may well benefit intestinal health and contribute to the increased growth rate observed for GOS-fed birds. GOS does not prevent *C. jejuni* colonization but may also lead to performance improvements by suppression of IL-17F under circumstances when *C. jejuni* colonization has been reported to be associated with bird health and productivity (Bull et al., 2008). These current studies were conducted in clean controlled biosecure facilities such that the birds would not be subject to exposure to endemic viral, bacterial and protozoal pathogens that are frequently encountered by commercial broiler chickens. Under these circumstances the impact of prebiotic priming of intestinal innate immunity may be of greater importance in field applications.

Consistent with previous reports we observed GOS-driven changes in the cecal microbiota of chickens featuring specific operational taxonomic units identifiable as lactobacilli (Hughes et al., 2017; Azcarate-Peril et al., 2018). Colonization by *C. jejuni* did not prevent the differential increase in abundance of Otu0017 (*L. johnsoniii*) associated previously with dietary GOS (Richards et al., 2019b). At 2dpi for the 6-dc birds Otu0002 (*L. crispatus*) and Otu0017 (*L. johnsoniii*) showed increases in abundance in association with the GOS-diet. However, Otu0017 (*L. johnsoniii*) remained an abundant member of the GOS + *Campylobacter* treatment group over the course of the experiment, and in contrast the relative abundance of Otu0002 (*L. crispatus*) fell such that it exhibited significantly greater abundance at 9 and 29 dpi in the *Campylobacter* treatment group fed the control diet. These data are consistent with *L. johnsonii* outcompeting *L. crispatus* in GOS-fed birds, and consistent with the hypothesis that *L. johnsonii* contributes to the stability of the innate immune response observed in GOS-fed birds. *L. johnsonii* is an established probiotic species that has been reported to improve growth performance, intestinal development, and act as competitive exclusion agent against bacterial pathogens in broiler chickens (La Ragione et al., 2004; Wang et al., 2017).

IL-17A responses to probiotics have been reported for *ex vivo* Peyer’s patch stimulated T cells derived from mice orally administered with lactic acid bacteria (*L. bulgaricus* or *Streptococcus thermophilus*). Over 7 days the stimulated T cells exhibited increases in the levels of IL-17 whilst IL-10 and Th2 IL-4 remained unchanged (Kamiya et al., 2016). In chickens transient IL-17 induction has been observed during the natural development the intestinal microbiota (Crhanova et al., 2011). In the absence of IL-22, pro-inflammatory Th17 induction did not result in intestinal damage but upon *Salmonella* Enteritidis challenge tissue damage was observed as a result of a Th17 response that features the cytokines IL-17 and IL-22.

## Conclusion

In conclusion the data support the contention that GOS diet-induced microbiota shifts can: (1) Improve the growth rate of broiler chickens independent of *C. jejuni* colonization. (2) Maintain ileal and cecal IL-17A transcription that can positively influence gut health in the presence of *C. jejuni*. (3) Suppress IL-17F expression arising as a consequence *C. jejuni* colonization that has the potential to impair gut integrity and health.

## Data Availability Statement

All 16S rDNA sequence data originally reported here is available at under accessions SRR10059315 to SRR10059356 in NCBI SRA study SRP133552. All other 16S rDNA sequences reported in this study are also available from NCBI SRA study SRP133552. Raw zootechnical and qPCR gene expression data is available from https://github.com/PJRichards/lafontaine_campy_gos.

## Ethics Statement

Experiments involving the use of birds were subjected to approval process under National Guidelines by the United Kingdom Home Office. Work on this project was approved under United Kingdom Government Home Office Project Licensing ASPA 86. The project license has been reviewed and approved by the University Ethics Committee prior to submission to the Home Office, which includes the scrutiny of animal welfare, ethics and handling.

## Author Contributions

NF contributed to the study design. NF and IC conceived and designed the experiments. GF, PR, PC, PO’K, NG, NC, and IC performed the experiments. GF, PR, PC, and IC analyzed the data and wrote the manuscript. All authors approved the final manuscript for publication.

## Conflict of Interest

NF is employed by Dairy Crest Ltd. The remaining authors declare that the research was conducted in the absence of any conflict of interest. The authors declare that this study received funding from Saputo Dairy UK. The funder had the following involvement with the study: NF contributed to the study design.

## Footnotes

1 https://github.com/mothur/mothur

## Supplementary Material

FIGURE S1 Rarefaction curves indicating sampling efficiency of cecal bacterial communities. Collectors curved were constructed for 16S rDNA sequences curated using mothur pipeline. Communities are presented by cohort as indicated by text in strip at the top of each panel. The colors indicate the rarefaction curves for individual bird in each cohort (key inset).

FIGURE S2 GOS responsive OTUs in age-matched broilers. Discriminative OTUs were identified using LEfSE between 6-dc challenged birds: Treatments *Campylobacter* and GOS + *Campylobacter* **(A)**, 20-dc challenged birds: Treatments *Campylobacter* and GOS + *Campylobacter* **(B)**, and 6-dc *Campylobacter*-challenged and GOS mock-challenged control birds: Treatments *Campylobacter* and GOS **(C)**. Comparisons of OTU relative abundance were made between age-matched cohorts that were rarefied to include only OTU ≥ 10 reads. For clarity only OTU with *p* < 0.05 for the embedded Kruskal Wallis ANOVA test and LDA (log_10_) > 2 are reported (Mothur defaults).

## Abbreviations

*C. jejuni*: *Campylobacter jejuni*
CFU: colony forming unit
FCR: feed conversion ratio
GoI: gene of interest
GOS: galacto-oligosaccharide
GIT: gastro-intestinal tract
IL: interleukin.
