# Supplementary figures and images for: Prebiotic Driven Increases in IL-17A Do Not Prevent Campylobacter jejuni Colonization of Chickens
Source: Front Microbiol. 2020 Jan 14;10:3030. doi: 10.3389/fmicb.2019.03030 (PMC6972505; doi:10.3389/fmicb.2019.03030)

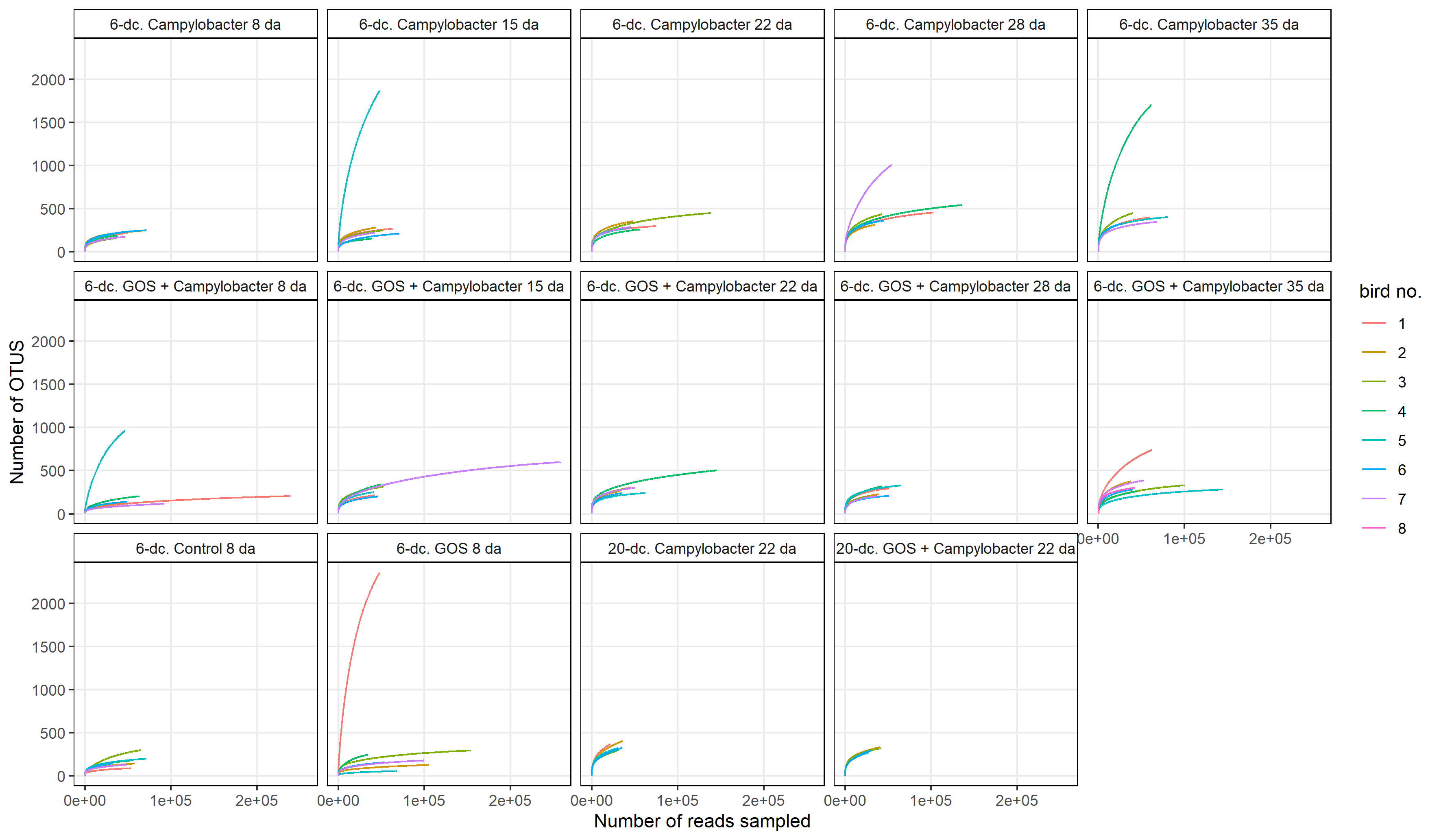

Supplement: FIGURE S1 — Rarefaction curves indicating sampling efficiency of cecal bacterial communities. Collectors curved were constructed for 16S rDNA sequences curated using mothur pipeline. Communities are presented by cohort as indicated by text in strip at the top of each panel. The colors indicate the rarefaction curves for individual bird in each cohort (key inset). [file Image_1.TIFF]

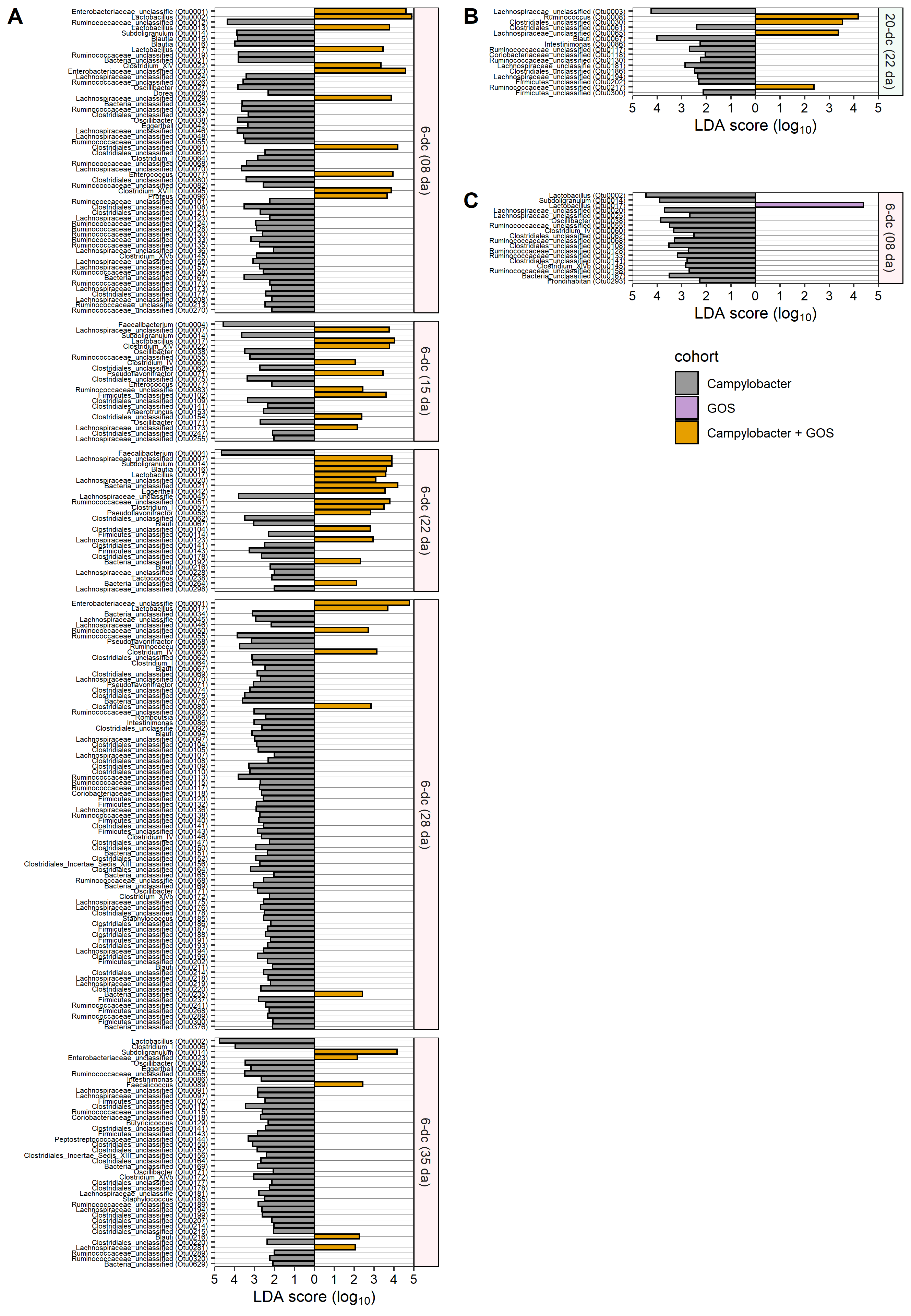

Supplement: FIGURE S2 — GOS responsive OTUs in age-matched broilers. Discriminative OTUs were identified using LEfSE between 6-dc challenged birds: Treatments Campylobacter and GOS + Campylobacter (A), 20-dc challenged birds: Treatments Campylobacter and GOS + Campylobacter (B), and 6-dc Campylobacter-challenged and GOS mock-challenged control birds: Treatments Campylobacter and GOS (C). Comparisons of OTU relative abundance were made between age-matched cohorts that were rarefied to include only OTU ≥ 10 reads. For clarity only OTU with p < 0.05 for the embedded Kruskal Wallis ANOVA test and LDA (log10) > 2 are reported (Mothur defaults). [file Image_2.TIFF]
